# More real-world evidence on the impact of emicizumab

**DOI:** 10.1016/j.rpth.2025.103188

**Published:** 2025-09-18

**Authors:** Jerome Teitel

**Affiliations:** Division of Hematology and Oncology, St. Michael’s Hospital, and University of Toronto, Toronto, Ontario, Canada

In this issue of *Research and Practice in Thrombosis and Haemostasis*, Wall et al. [1] report the results of a 3-year study on UK patients with severe hemophilia A (SHA) without inhibitors who were given the choice of switching to emicizumab for prophylaxis or remaining on factor (F)VIII concentrate [1]. Since access and cost were not barriers to treatment choice, this national cohort study provides insight into the acceptance of emicizumab by patients and their health care teams and data on how their outcomes compared with outcomes of those who chose to remain on conventional FVIII prophylaxis.

Emicizumab is a FVIII mimetic bispecific antibody that is administered by infrequent subcutaneous injection [2]. It was the first alternative to replacement therapy for prophylaxis in patients with SHA with or without FVIII inhibitors. The efficacy and wide safety margin of emicizumab were documented in a series of phase 3 clinical trials, the “HAVEN” program [3]. Emicizumab has been eagerly and widely adopted by people with hemophilia and by health care professionals. Now, nearly 8 years after its first approval, it is important to document its medium-term and long-term outcomes with real-world evidence.

The authors report that 40.3% of the 1535 eligible patients with SHA chose to switch from FVIII prophylaxis to emicizumab. The study accrual period was sufficiently long that it is likely that almost all patients who were motivated to make the switch had the opportunity to do so. The fact that fewer than half made this choice suggests a high degree of patient satisfaction with their FVIII regimens.

Reasons for not switching were not reported, but it is possible to speculate about some of them. FVIII in sufficient dosage can fully correct the hemostatic defect of hemophilia, and the degree of correction can be easily individualized and predicted; this is an attractive feature for many patients, such as those who engage in vigorous physical and athletic activity, who can be assured of peak protective levels of FVIII when they are at greatest risk of bleeding. In contrast, emicizumab corrects hemostasis only partially, and its cofactor potency relative to FVIII cannot be estimated with confidence [4,5]. Another group that may be reluctant to withdraw from FVIII is patients with previous inhibitors, who may fear losing tolerance and experiencing inhibitor recurrence. The authors do not state how many of the nonswitchers in the UK cohort had a history of an inhibitor. In those who did switch to emicizumab, there were 84 with a previous inhibitor, and inhibitors recurred in 9.5% of them over the course of the study. Surprisingly, none of these recurrences occurred in the context of re-exposure to FVIII. However, in most of them (5/8), there was evidence of residual low-level inhibitor activity, suggesting that before they switched, FVIII had been maintaining immune tolerance as well as providing prophylaxis.

The majority (78.2%) of emicizumab-treated patients reported by Wall et al. [1] had been on standard half-life (SHL) FVIII concentrates before switching, and the rest were on extended half-life (EHL) products. It is not stated whether the SHL FVIII–treated patients were offered the option of switching to an EHL FVIII concentrate as an alternative to emicizumab. However, it is unlikely that the minor pharmacokinetic advantage of conventional EHL concentrates would have persuaded many patients to remain on FVIII. In Canada, the large majority of patients with SHA who were on prophylaxis prior to the introduction of emicizumab were using EHL concentrates. Data from our national bleeding disorders registry accessed in August 2025 show that 58% of patients with SHA (578/973) chose to switch to emicizumab (unpublished), an even higher percentage than the 40.3% who switched in the UK cohort. Efanesoctocog, a FVIII concentrate with terminal half-life of approximately 4-fold greater than SHL FVIII products, has become available since the termination of the UK study [6]. Once-weekly dosing with efanesoctocog provides excellent protection from bleeding and maintains FVIII levels in the normal or mild hemophilia range for the entire week [7]. If it is approved in the United Kingdom in the near future and is made available to patients with SHA, it will be interesting to observe whether any patients currently on emicizumab will consider switching back to FVIII, given the option of prophylaxis with an authentic replacement product that requires substantially fewer infusions.

Adherence to prescribed treatment is a challenge in severe hemophilia, given the need for frequent self-infusion to maintain effective prophylaxis [8,9]. Just under than half of the nonswitchers in the UK study (47%) were compliant with their prescribed FVIII concentrate regimen, based on product utilization as recorded in web-based diaries. In contrast, poor compliance was reported as an adverse event in only 1 of 618 patients on emicizumab. This is reassuring, but in any case the notion of compliance has a different connotation with respect to emicizumab, a drug with a half-life of 29 days [10], than it does for FVIII concentrates, with half-lives varying from approximately 12 hours for SHL products to approximately 45 hours for efanesoctocog. Emicizumab achieves stable plasma levels, such that delayed or even missed doses have considerably less clinical significance than they do for FVIII concentrates.

Outcome measures in hemophilia have become more problematic as the efficacy of treatment has progressively improved. The traditional outcome of annualized bleeding rate loses its sensitivity as these rates asymptotically approach zero with current prophylaxis regimens. Payers may be willing to reimburse such products only if they demonstrate meaningful improvements in other outcomes that yield an acceptable incremental cost-effectiveness ratio, such as quality of life [11]. The primary efficacy outcome in the study by Wall et al. [1], annualized treated bleeding rate, decreased after switching from FVIII prophylaxis, although from already very low baseline levels. The nearly 50% increase in the proportion of patients reporting zero bleeds was particularly impressive. A caveat is that bleeding rate is a patient-reported outcome, and the distinction between symptoms of bleeding and of structural or inflammatory pain (especially in joints) is not always clear to the patient or his health care professional [12,13]. As a result, the designation of a breakthrough bleed requiring FVIII infusion is affected to some extent by patients’ confidence in their prophylaxis products. On the other hand, the resolution of 82.1% of target joints after switching to emicizumab is an objective and impressive indicator of the efficacy of emicizumab. Lack of efficacy was reported in only 2 patients. Another patient experienced a fatal intracerebral hemorrhage. However, as his plasma emicizumab level was well within the therapeutic range at the time, lack of efficacy or drug resistance was likely not causative.

Emicizumab has a good safety record and is generally well tolerated [14,15]. In the UK cohort, only 31 adverse events were reported in 28 individuals (4.5% of those at risk). Five of the events were deaths, all of them judged as unrelated to treatment. Only 14 patients (2.2%) discontinued emicizumab, but it appears that this was due to a drug-related adverse effect in only 8 cases (including 3 systemic reactions and 1 antidrug antibody).

Thrombosis is an event of special concern in nonreplacement hemostatic therapy for hemophilia. Thrombotic events were seen in the HAVEN program, including the unexpected occurrence of thrombotic microangiopathy [16]. Although the majority of these cases occurred during concomitant therapy with activated prothrombin complex concentrate, postlicensure safety surveillance studies in Europe [17] and the United States [18] indicate that the thrombotic complication rate of emicizumab exceeds that of FVIII concentrates. In the UK cohort report, only 1 thrombotic adverse event was reported, an unusual case of bilateral renal infarcts. This occurred on the background of renal artery disease (fibromuscular dysplasia), but as it occurred only after the patient discontinued FVIII and switched to emicizumab, the investigator appropriately considered the drug to be contributory. It is possible that there were additional contributory factors to this unusual event, such as other drugs or a thrombophilic abnormality.

Over the last 70 years, successive innovations in replacement therapy, from cryoprecipitate to gene insertion, have progressively transformed hemophilia from a crippling or fatal affliction into a manageable disorder compatible with near normal longevity and quality of life [19]. Emicizumab, the first subcutaneously administered “non-replacement” agent for continuous prophylaxis, represents an important landmark in this history. Other subcutaneous agents with different mechanisms of action, the so-called “rebalancing agents,” are already in use or under development [20,21]. The research studies that led to the approval of emicizumab constituted an exemplary clinical trial program. Now it is the responsibility of all those in the hemophilia community, patients and health care professionals, to collaborate in follow-up studies to assess its long-term safety and efficacy outcomes. National databases such as that maintained by the United Kingdom Haemophilia Centre Doctors’ Organisation are an invaluable tool in this research, and the report by Wall et al. [1] is a welcome contribution.
